# The role of Phosphodiesterase-1 and its natural product inhibitors in Alzheimer’s disease: A review

**DOI:** 10.3389/fphar.2022.1070677

**Published:** 2022-12-21

**Authors:** Nazir Ahmad, Kaisun Nesa Lesa, Ari Sudarmanto, Nanang Fakhrudin, Zullies Ikawati

**Affiliations:** ^1^ Department of Pharmacology and Clinical Pharmacy, Faculty of Pharmacy, Universitas Gadjah Mada, Sekip Utara, Yogyakarta, Indonesia; ^2^ Department of Food and Agricultural Product Technology, Faculty of Agricultural Technology, Universitas Gadjah Mada, Yogyakarta, Indonesia; ^3^ Department of Pharmaceutical Chemistry, Faculty of Pharmacy, Universitas Gadjah Mada, Sekip Utara, Yogyakarta, Indonesia; ^4^ Department of Pharmaceutical Biology, Faculty of Pharmacy, Universitas Gadjah Mada, Sekip Utara, Yogyakarta, Indonesia; ^5^ Medicinal Plants and Natural Products Research Center, Faculty of Pharmacy, Universitas Gadjah Mada, Sekip Utara, Yogyakarta, Indonesia

**Keywords:** phosphodiesterases, memory disorder, medicinal plant, secondary metabolite, memory enhancement

## Abstract

Phosphodiesterase-1 (PDE1) is a versatile enzyme that has surprisingly received considerable attention as a possible therapeutic target in Alzheimer’s disease (AD) because it maintains the homeostasis of 3ʹ,5ʹ-cyclic adenosine monophosphate (cAMP) and 3ʹ,5ʹ-cyclic guanosine monophosphate (cGMP) in the brain. 3ʹ,5ʹ-cyclic adenosine monophosphate and 3ʹ,5ʹ-cyclic guanosine monophosphate are the two key second messengers that regulate a broad range of intracellular processes and neurocognitive functions, specifically memory and cognition, associated with Alzheimer’s disease. However, the lack of available selective drugs on the market poses challenges to identifying the beneficial effects of natural products. The present review focuses on Phosphodiesterase-1 and its isoforms, splicing variants, location, distribution, and function; the role of Phosphodiesterase-1 inhibitors in Alzheimer’s disease; and the use of vinpocetine and natural products as specific Phosphodiesterase-1 inhibitors. Moreover, it aims to provide ongoing updates, identify research gaps, and present future perspectives. This review indicates the potential role of Phosphodiesterase-1 inhibitors in the treatment of neurodegenerative disorders, such as Alzheimer’s disease. Certain clinical trials on the alleviation of Alzheimer’s disease in patients are still in progress. Among *de novo* outcomes, the employment of Phosphodiesterase-1 inhibitors to treat Alzheimer’s disease is an important advancement given the absence of particular therapies in the pipeline for this highly prevalent disease. To sum up, Phosphodiesterase-1 inhibition has been specifically proposed as a critical therapeutic approach for Alzheimer’s disease. This study provides a comprehensive review on the biological and pharmacological aspects of Phosphodiesterase-1, its role on the Alzheimer’s diseases and its significance as Alzheimer’s disease therapeutic target in drug discovery from natural products. This review will help clinical trials and scientific research exploring new entities for the treatment and prevention of Alzheimer’s disease.

## 1 Introduction

**FIGURE 1:**
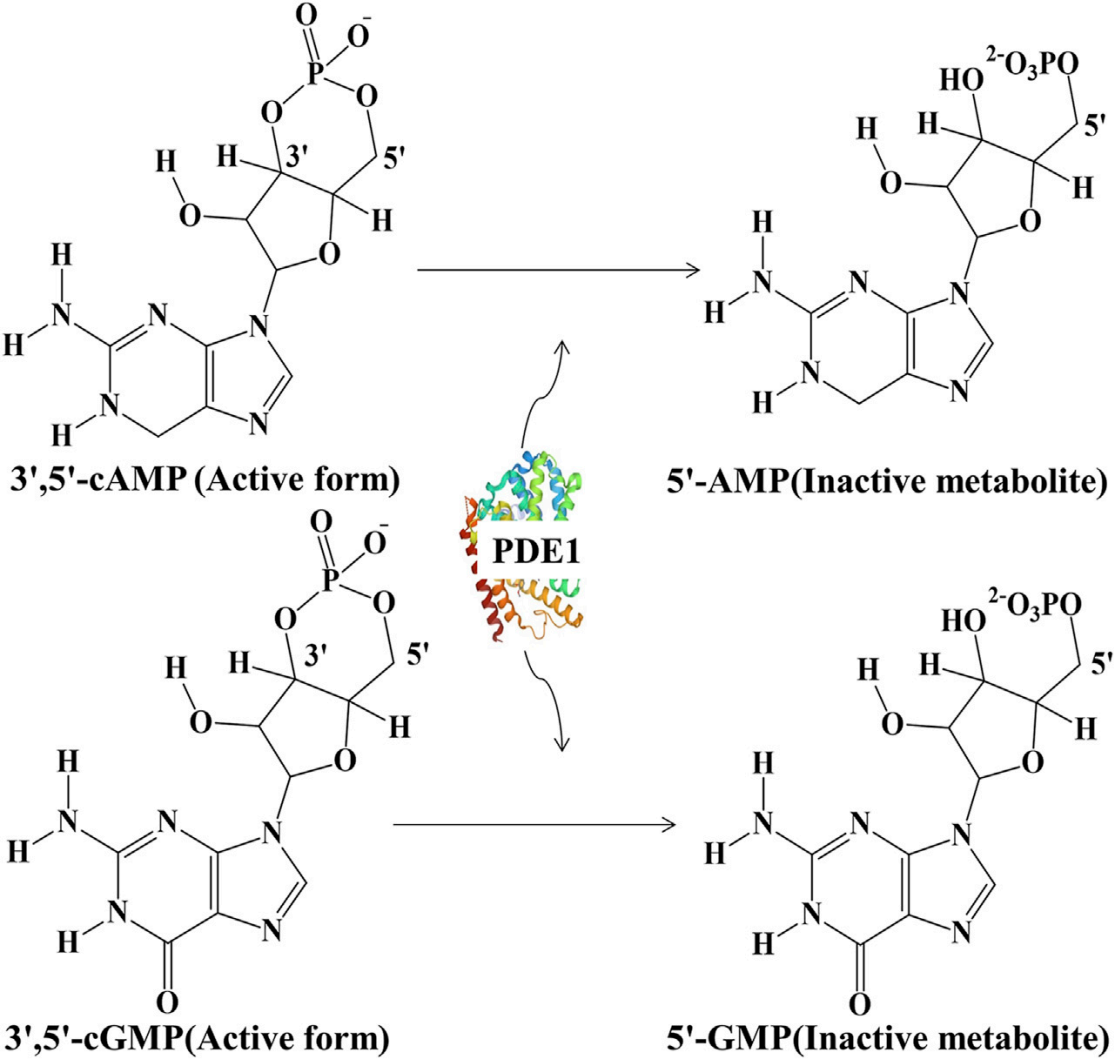
Hydrolysis of active 3ʹ,5ʹ-cAMP into inactive 5ʹ-cAMP and active 3ʹ,5ʹ-cGMP into inactive 5ʹ-cGMP by PDE1.

Phosphodiesterases (PDEs) constitute a large family of phosphohydrolytic enzymes; they were discovered approximately half a century ago and have since become a focal point of today’s research in multiple fields (Blokland et al., 2019). PDEs hydrolyze the intracellular second messengers 3ʹ,5ʹ-cyclic adenosine monophosphate (cAMP) and 3ʹ,5ʹ-cyclic guanosine monophosphate (cGMP) into their inactive metabolites 5ʹ-cAMP and 5ʹ-cGMP, respectively (Figure 1) (Tian et al., 2014). They are classified into 11 distinct families that comprise 21 separate genes and more than 100 gene variants (Samidurai et al., 2021). However, they differ in affinity for cAMP/cGMP. For example, PDEs 1–3, 10, and 11 have affinity for both cyclic nucleotides; PDEs 4, 7, 8 are cAMP-specific; and PDEs 5, 6, and 9 are cGMP hydrolytic enzymes (Heckman et al., 2017). Isoforms of all PDEs are widespread in several organs with sole functional activities. Their activation is linked to pathophysiology, and their inactivation or inhibition is associated with disease recovery. Moreover, several clinical implications of the inhibition of PDEs have become appreciated in many areas after the historic discovery of the vasodilating effect of nitric oxide (NO) on the cardiovascular system (Samidurai et al., 2021). PDEs are emerging as new cellular molecular targets for the discovery of novel pharmaceutical entities to treat neurodegenerative diseases (NDDs), such as Alzheimerʹs disease (AD). Supplementary well-planned clinical studies are needed to ascertain the efficacy and safety of PDE inhibitors in individuals with AD (Nabavi et al., 2019). Among all members of the PDE superfamily of enzymes, PDE1 has been found to be highly expressed in the brain (Helmi et al., 2020b) and is considered to be responsible for AD development *via* the mechanism of cAMP/cGMP downregulation in neuronal cells (Ribaudo et al., 2021).

PDE1, a dual-substrate PDE enzyme, hydrolyzes 3ʹ,5ʹ-cAMP and 3ʹ,5ʹ-cGMP, which are synthesized by adenylyl cyclase (AC) and guanylyl cyclase (GC), respectively, into inactive metabolites. Subsequently, these inactive products are expelled from cells under the mediation of multidrug resistance-associated protein-4 (Chen et al., 2019). PDE1 activation is linked to Ca^2+^, and an endogenous protein factor enhances the sensitivity of PDE1 to Ca^2+^. At normal concentrations, Ca^2+^ and calmodulin (CaM) form the Ca^2+^–CaM complex, thereby stimulating PDE1 (Kakkar et al., 1999). PDE1 is highly expressed in distinct areas of the brain, where its key role is to regulate cognitive functions (Helmi et al., 2020a). Both cyclic nucleotides are essential in brain cells for neurodevelopment, neuroplasticity, and consequently enhancing learning and memory (Delhaye and Bardoni, 2021). Recently, three PDE1 genes have been identified in the central nervous system (CNS), namely, PDE1A, PDE1B, and PDE1C. Among these three genes, PDE1B has been found to be responsible for >90% of brain activity accompanying learning and memory. PDE1B is promising drug target for the treatment of NDDs given this feature (Shy and Gaurav, 2021a).

NDDs are CNS function-disrupting diseases that affect the brain, spinal cord, and peripheral nerves. The most common NDD is AD, which is a chronic irreversible disease with gradual onset and deterioration as it progresses (Javaid et al., 2020). Patients with AD present memory loss, decreased learning ability, and odd behavior (Saleem et al., 2021). The primary pathophysiology of AD is linked to the deposition of amyloid β (Aβ) plaques and the aggregation of tau neurofibrillary tangles (Liu et al., 2017). Memory is one of the popular indexes for diagnosing cognition in AD. Its loss, coupled with abnormalities of the hippocampus and prefrontal lobe, is clinically seen in patients with AD. Likewise, decreasing concentrations of cAMP/cGMP and brain-derived neurotrophic factor (BDNF) in the brain have been found to be associated with AD progression. Previous studies have demonstrated that cAMP/cGMP is hydrolyzed by the enzyme PDE1, a crucial target in AD, and causes cognitive dysfunction (Helmi et al., 2021). Epidemiologically, AD is a major health burden worldwide (Dubey et al., 2020), and its current annual cost is estimated to be US $1.3 trillion, a figure set to double by 2030; additional contemporary cases of AD have been reported (World Alzheimer Report 2022; https://www.alzint.org/about/dementia-facts-figures/). Before the end of 2050, 1 out of 85 persons is anticipated to face AD, which is the most common type of dementia that chiefly affects elderly individuals (Saleem et al., 2021). Therefore, in the last several years, the investigation of cognitive decline has attracted increasing attention (Martínez et al., 2021). Indeed, modern lifestyle has not only made life easy but also elevated the risk of chronic diseases, eventually exacerbating the excessive consumption of pharmaceuticals that are sometimes known as nootropic agents or cognition enhancers, e.g., vinpocetine. Although vinpocetine selectively inhibits PDE1, it exerts undesirable side effects (Dubey et al., 2020). Chemical medicine therapy is also sometimes linked to some unwanted effects that impede the achievement of therapeutic efficacy. Therefore, the discovery of novel and alternative therapeutic entities from natural products, which may have the best features of effectiveness and safety, is needed.

Natural products for primary healthcare are catching the attention of the masses and multiple healthcare staff. A 2010 WHO report indicated that one third of the population uses different plant products to improve their health. The cost of hospital visits, chemical drug resistance, and the poor efficacy and scarcity of medicine have led people to seek accessible herbal medicine treatment (Chowdhury et al., 2022). The various ethnopharmacological implications of traditional plants, have to be evaluated scientifically to enable their reasonable and safe use (Rauf et al., 2015). For example, a recent investigation confirmed that in a mouse model with scopolamine-induced memory impairment, *Caesalpinia sappan* L. enhanced cognition by targeting PDE1 (Helmi et al., 2021). However, *C. sappan* L. extract cannot be recommended for the market because of its limited clinical data. The other plant extracts mentioned in this review encounter the same problem. Therefore, PDE1 inhibitors on the market remain lacking (Helmi et al., 2020a). The dearth of effective therapies and fabrication of new strategies for AD remains a challenging issue (García-Osta et al., 2012).

The aim of the current review is to highlight PDE1 and its subtypes, location, and function and the relationships between the activation of PDE1 and the pathophysiology of cognitive decline, between the inhibition of PDE1 and AD, and between the inhibition of PDE1 and natural products. It also aims to provide a report on the latest research on the treatment of AD and the gaps in the research on AD.

## 2 Methodology

We reviewed more than 200 scientific papers from different literature sources, such as PubMed (www.ncbi.nlm.nih.gov/pubmed/), Google Scholar (https://scholar.google.com.pk/), and Scopus (www.scopus.com). Only 83 papers were found suitable for this review. For this review, we considered four articles that were published in the 19th and 20th centuries, and the remaining articles were recently published from 2012 to 2022. A systematic scheme was adopted for the review of PDE1 and its isoforms, activation, and inhibition in the brain by taking into account the details of the *in vitro* and *in vivo* data available. The following keywords were used to search databases: “phosphodiesterase 1,” “phosphodiesterase 1” AND “cognition,” “pde-1” AND “cognition,” “health benefits of plant,” “PDE1 AND plant extract AND cognition,” “PDE1 and pathophysiology of Alzheimer,” “ca AND pde1,” “PDE1 inhibitors, Alzheimer,” “vinpocetine stability,” “citrus fruits, Alzheimer,” “*Caesalpinia sappan*,” “*Heterophragma adenophyllum* Seem,” “*Claviceps purpurea*,” “*Periandra dulcare* Mart.,” “*Nelumbo nucifera*,” and “*Vinca rosea*,” “PDE1, cognitive enhancer,” “Herbs, phosphodiesterase, Alzheimerʹs disease,” “PDE1 inhibitor, pharmacognosy, *Caesalpinia sappan* L.,” “PDE1, cognitive enhancer,” “*Heterophragma adenophyllum* Seem,” “*Claviceps purpurea*, fungus,” “*Claviceps purpurea* (ergot), ergot alkaloids,” “*Periandra dulcare* Mart.,” “*Periandra dulcare* Mart., PDE,” “*Nelumbo nucifera*,” “neferine alleviates memory, cognitive dysfunction,” “lotus, horticultural plants,” “*Vinca rosea*, natural plants, brain,” “citrus fruits, brain,” and “neuroprotective, citrus fruit, Alzheimer.” All structures were drawn by using ChemDraw Ultra 12.0 software.

## 3 History, distribution, types, and functional properties of PDE1

### 3.1 History of PDE1

PDE1 has a sound historic background. The first study on PDE1 was performed several years ago in 1968. This work illustrated the role of PDEs in the kidney, wherein PDEs regulated cAMP signaling inside cells. Another study published several years later indicated that xanthine derivatives in fat cells inhibited the activity of PDEs. A 1972 study further found two distinct classes of PDEs in amoebas. This finding was thought as the first evidence for further PDE classification. Afterward, many subtypes were recognized on the basis of similarities in structures and enzymatic behaviors (Enomoto et al., 2019). Eleven PDE families (PDE1–11) have been recently identified in mammals (Blokland et al., 2019). However, this review focuses only on PDE1 because of its high expression in the brain.

### 3.2 Distribution of PDE1

Enzymes are biocatalysts that catalyze biological reactions and are crucial components of cellular metabolism (González-Rodríguez et al., 2022). PDEs constitute a large family of enzymes that convert cyclic nucleotides into their monophosphate isoforms (Nabavi et al., 2019). Among all other PDEs, PDE1 is highly expressed in AD brain regions, such as the hippocampus, frontal cortex, temporal cortex, parietal cortex, and stratum (Helmi et al., 2020a), and is specifically expressed in Purkinje neurons in the cerebellum (García-Osta et al., 2012). PDE1 is a wide spread enzyme in body. However, it is highly expressed in brain. Therefore, PDE1 inhibitors can be considered as potentially useful for AD treatment because they have considerable selective PDE1 inhibitory action, thereby strengthening synaptic functions. Many studies are available in which this enzyme was being targeted for curing cognitive dysfunction and AD (Helmi et al., 2020a, 2021; Delhaye and Bardoni, 2021). PDE1 inhibitors are considered as a practicable choice to reverse dementia and AD symptoms (Shekarian et al., 2020a).

### 3.3 Types of PDE1

PDE1 has three isoforms: PDE1A, PDE1B, and PDE1C. PDE1A is highly expressed in the cornu ammonis (CA1, CA2, and CA3) of the hippocampus and the fifth to sixth layers of the cortex. Correspondingly, PDE1B has been found to have the highest expression in specific areas of the brain (Enomoto et al., 2019), such as the striatum and dentate gyrus of the hippocampus (Betolngar et al., 2019), particularly in subsets of Purkinje cells (García-Osta et al., 2012). PDE1C is often synthesized in the cerebellum (Betolngar et al., 2019). Although all PDE1 genes have high affinity for cAMP and cGMP, their tissue distribution patterns are quite different (García-Osta et al., 2012). Moreover, more than 90% of the total brain activity depends on PDE1B, which is linked to memory and learning processes, thus making this isotype a desired drug target for protection against AD (Shy and Gaurav, 2021b). In addition to the brain, PDE1A and PDE1C are highly expressed in peripheral tissues (Table 1), such as the lung, bladder, heart, kidney, and thyroid, where they play different key roles (Enomoto et al., 2019).

**TABLE 1:** Expression, localization, and function of PDE1 isoforms and their gene variants.

Isoform/Gene	Gene variant	Expression	Intracellular localization	Activated by	Target hydrolysis	Function	References
PDE1A	—	Brain, sperm, smooth muscle, lung, heart	Predominantly cytosolic	Ca2+/CaM	cAMP < cGMP	Proliferation activates p27Kip1 and regulates the cell cycle, sperm function, nitrate tolerance, vascular contraction of mesenteric arteries, autosomal dominant polycystic kidney disease, and myocardial-alpha-crystallin B chain	Samidurai et al. (2021)
	PDE1A1	Lung, heart	Predominantly cytosolic	Ca2+/CaM	cAMP < cGMP	Same as PDE1A	Menniti et al. (2006)
	PDE1A2	Brain	Predominantly cytosolic	Ca2+/CaM	cAMP < cGMP	Parkinson’s Disease	Therapies, (2011)
	PDE1A3	Multiple human tissues	Cytosolic	Ca2+/CaM	cAMP < cGMP	Hypertension	Laursen et al. (2017)
PDE1B	—	Brain	Cytosolic	Ca2+/CaM	cAMP < cGMP	Cognitive-enhancing effect	Shy and Gaurav, (2021b)
	PDE1B1	Brain, neurons, smooth muscle, heart, skeletal muscle, lymphocytesy	Cytosolic	Ca2+/CaM	cAMP < cGMP	AD, dopaminergic function, neuronal learning, apoptosis induction in leukemia cells	Samidurai et al. (2021)
	PDE1B2	Lymphocytes, macrophages	Cytosolic	Ca2+/CaM	cAMP < cGMP	Differentiation of monocytes–macrophages	Jia et al. (2020)
PDE1C	PDE1C1	Brain, testis, heart	—	Ca2+/CaM	cAMP = cGMP	Age-related cAMP signaling regulation, cardiomyocyte apoptosis	Nakano et al. (2017)
	PDE1C2	Olfactory epithelium	Cytosolic	Ca2+/CaM	cAMP = cGMP	Odorant stimulation	Moon et al. (2005)
	PDE1C3	Newborn and adult aortas	Cytosolic	Ca2+/CaM	cAMP = cGMP	Unknown	Omori and Kotera, (2007)
	PDE1C4/5	mRNA is present in the testes	—	Ca2+/CaM	cAMP = cGMP	Unknown	Yan et al. (1996)

### 3.4 Splicing variants of PDE1 isoforms

**FIGURE 2:**
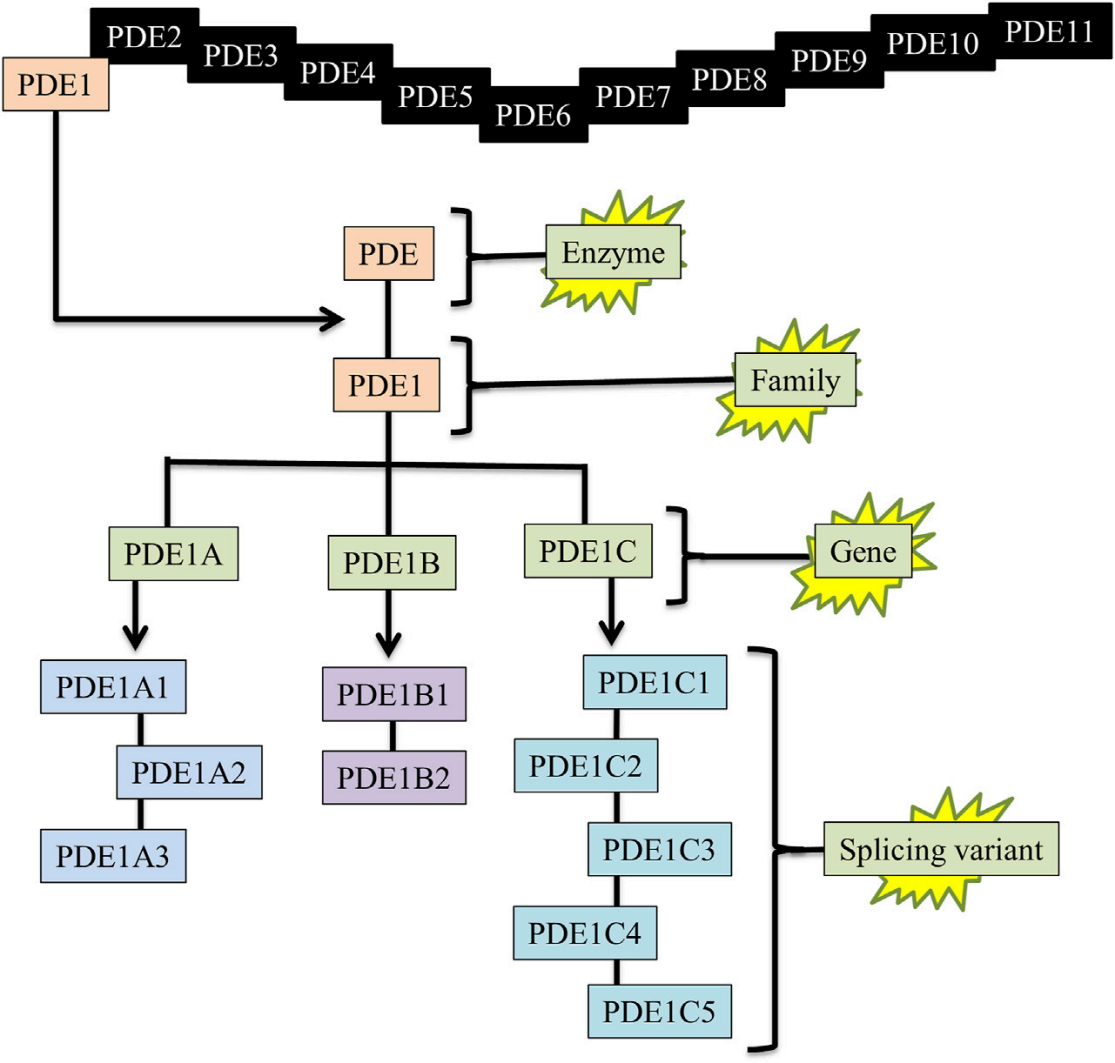
Enzyme, family, gene, and splicing variants of PDE1. PDE = Phosphodiesterase, PDE1 = Phosphodiesterase-1, PDE1A1 = (PDE enzyme, 1-family, A-gene, 1-splicing variant).

Splicing variants or gene variants enhance functional diversity and extend regulatory activities. Specifically, protein isoforms synthesized by splicing variants can have variations in catalytic abilities, protein-protein interlinkages, or subcellular localization. The splicing variants of PDE1 genes contribute to the regulation of normal physiological functions and pathological processes (Boldinova et al., 2019). Each PDE1 gene has multiple gene variants. PDE1A has three gene variants, e.g., PDE1A1, PDE1A2, and PDE1A3; PDE1B has two gene variants, e.g., PDE1B1 and PDE1B2; and PDE1C has five gene variants, e.g., PDE1C1, PDE1C2, PDE1C3, PDE1C4, and PDE1C5 (Figure 2) (Samidurai et al., 2021). The location and function of these splicing variants completely differ from each other.

### 3.5 Functional properties of PDE1

PDE1 and its isoforms produce inactive products from both second messengers. PDE1 isoforms, such as PDE1A and PDE1B, hydrolyze cGMP more than cAMP, whereas PDE1C targets cGMP and cAMP equally. Their activities are modulated by Ca^2+^/CaM. The lack of meaningful data on the functional complications of these specific isoforms of PDE1 and serious challenges in the selectivity of previous inhibitors have considerably decelerated (Menniti et al., 2006) and impeded the progress of research analyzing the precise role of each PDE1 isoform (Samidurai et al., 2021). Therefore, in this review, we highlight splicing variants to progress this field. A detailed explanation of PDE1 and its isoforms along with their gene variants is provided in Table 1.

In all PDE isoforms, the main variable regions are N- and C-terminal domains. Each member differs from other members in terms of their cellular or subcellular localization, which is the most important element for identifying the role of each PDE. Enzymatic activity is enhanced by Ca^2+^/CaM binding to the regulatory site present in the N-terminal region of PDE1. An intronic single nucleotide polymorphism in the PDE1C gene is linked to autism spectrum disorder (ASD). An inherited missense variant of the PDE1B gene has been found in ASD and schizophrenia. These results indicate the extreme need to modulate PDE1 gene expression by using pharmacological approaches to assess their influence on cognitive behavior in the population (Delhaye and Bardoni, 2021).

## 4 PDE1 and cAMP/PKA/CREB/BDNF and cGMP/PKG/CREB/BDNF pathways

The activation of the cAMP/PKA/CREB/BDNF and cGMP/PKG/CREB/BDNF pathways is crucial for neuroplasticity and memory function (Luo et al., 2017). The dual-substrate PDE1 impedes this pathway by hydrolyzing cAMP/cGMP and interfering with cognitive function (Helmi et al., 2021). In contrast, in human brains, the inhibition of highly expressed PDE1 protects against neurodegeneration (Xi et al., 2022). Certain stimuli trigger PDE1 activation. For example, any surge in Ca^2+^ augments the interaction between Ca^2+^ and CaM (Kogiso et al., 2020) (Enomoto et al., 2019). The catalytic subunits of PDE1 isozymes act as dimers, and every individual monomer possesses two CaM binding sites: a catalytic domain and an autoinhibitory subdomain. Ca^2+^/CaM attachment relieves autoinhibition (Francis and Corbin, 2013). Eukaryotic cells have multiple Ca^2+^ entry pathways, such as calcium channel opener second messengers (e.g., inositol 1,4,5-trisphosphate influences the reticulum Ca^2+^ storage. Alternatively, Ca^2+^ enters cells through voltage-gated Ca^2+^-channels or channels that are opened by different intracellular and extracellular messengers after complex formation activates the PDE1 enzyme (Goraya and Cooper, 2005).

**FIGURE 3:**
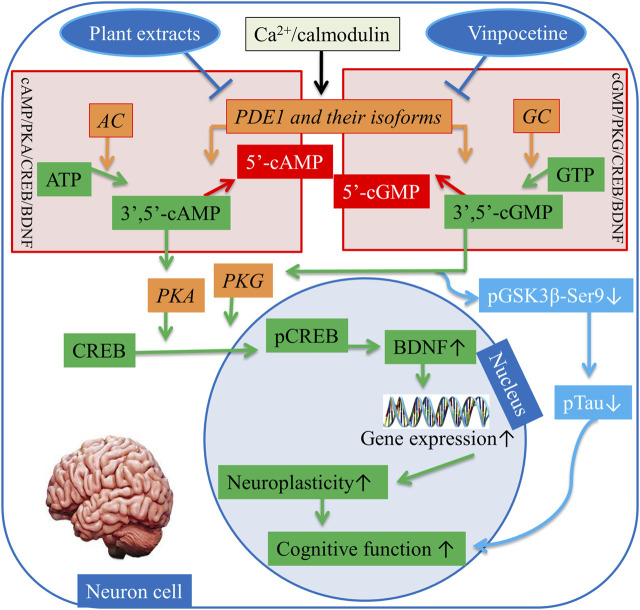
Regulation of the cAMP/PKA/CREB/BDNF and cGMP/PKG/CREB/BDNF pathways. AC = Adenylyl cyclase, GC = Guanylyl, PDE1 = Phosphodiesterase-1, ATP = Adenosine monophosphate, GTP = Adenosine guanosine monophosphate, 3ʹ,5ʹ-cAMP = 3ʹ,5ʹ-cyclic adenosine monophosphate, 5ʹ-cAMP = 5ʹ-cyclic adenosine monophosphate, PKA = Protein kinase A, pGSK3beta-Ser9 = Phosphorylated glycogen synthase kinase-3β-(Ser9), PKG = Protein kinase G, CREB = cAMP response element binding, pCREB = Phosphorylated cAMP response element binding, BDNF = Brain-derived neurotrophic factor. ↑, increase sign; ↓, decrease sign.

Inversely, upon the activation of G protein-coupled receptors in the brain, heterotrimeric G proteins activate AC in response to extracellular stimuli, whereas Ca^2+^ activates soluble AC directly. Similarly, transmembrane GC is activated by C-type natriuretic peptide, and soluble GC is activated by NO in the brain. Hence, AC and GC activation triggers a signaling cascade in neuron cells. AC catalyzes adenosine triphosphate (ATP) and synthesizes cAMP. In a similar fashion, GC catalyzes guanylyl triphosphate (GTP) and synthesizes cGMP (Francis and Corbin, 2013). cAMP and cGMP together regulate various cellular pathways by phosphorylating protein kinase A (PKA) and PKG, respectively. Ultimately, PKA and PKG activate transcriptional proteins, e.g., cAMP response element-binding protein (CREB) into phosphorylated CREB (pCREB), which is involved in learning and memory (Delhaye and Bardoni, 2021). pCREB is a nucleus protein that initiates gene transcription directly by interfering with gene promoters in the nucleus, where it “switches on” the expression of several genes, such as BDNF. BDNF is a neurotropic factor that has been investigated for its involvement with the proliferation, differentiation, and plasticity of neurons to regulate and maintain cognitive function, particularly learning and memory (Wu et al., 2018a; Helmi et al., 2021). Moreover, cGMP directly inactivates a kinase enzyme known as phosphorylated glycogen synthase kinase-3β-(Ser9) (pGSK3β-Ser9), and in turn phosphosphorylation of Tau (pTau) decreases (García-Osta et al., 2012). The reduction in the phosphorylation of pTau elicits the protection of neurons and eventually enhances cognitive function (Jiang et al., 2022). Vinpocetine has been used for a long time to treat dementia by selectively inhibiting PDE1 (Shekarian et al., 2020a; Dubey et al., 2020). Given the above mechanism, plant extracts also hinder PDE1 and its isoforms’ activities in the brain in a manner similar to specific inhibition by vinpocetine (Zang et al., 2021). Thus, activating the cAMP/PKA/CREB/BDNF and cGMP/PKG/CREB/BDNF signaling pathways can alleviate AD symptoms by restoring synaptic function (Figure 3).

## 5 Activation of PDE and pathophysiology of cognitive decline

**FIGURE 4:**
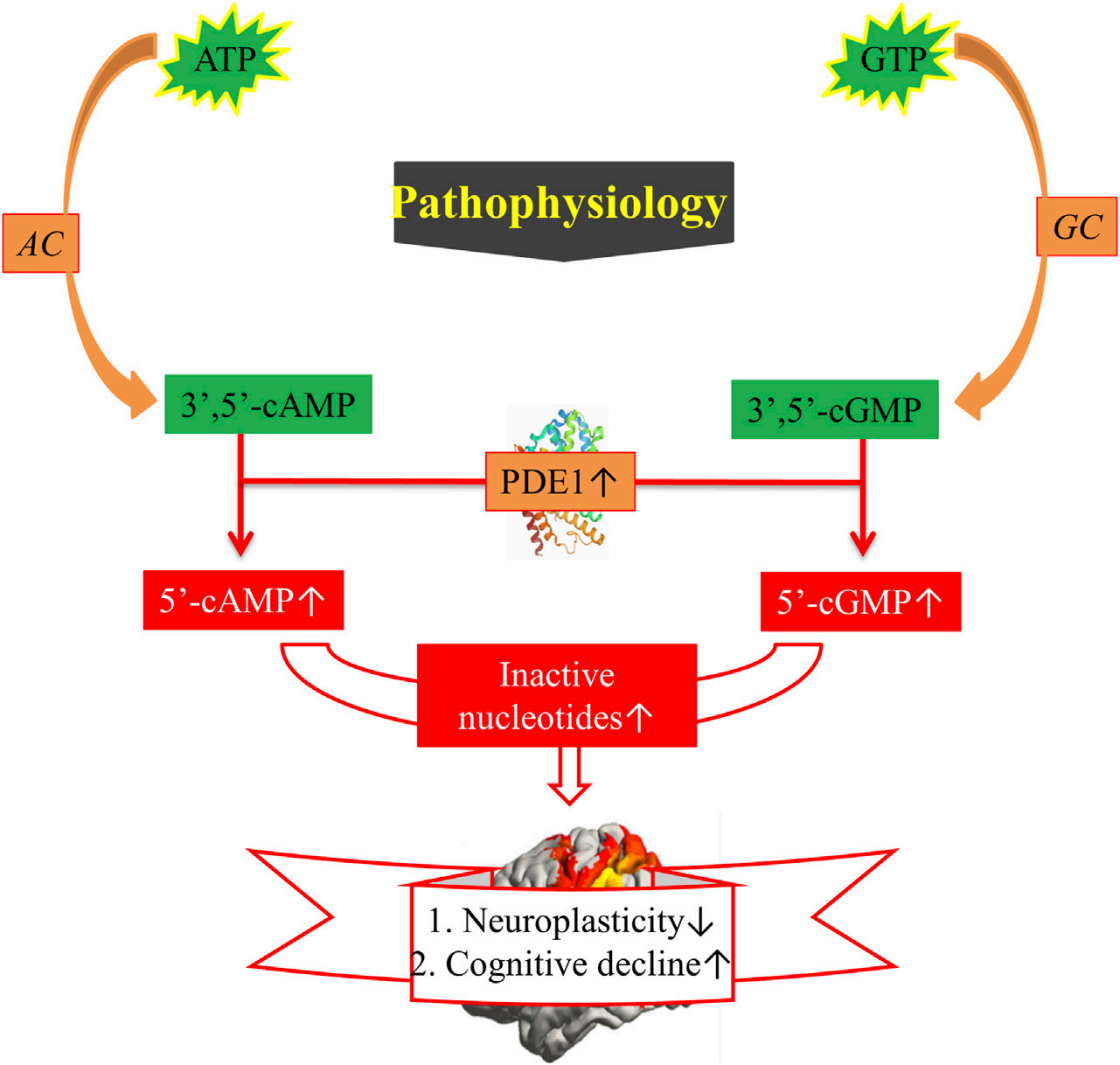
Pathophysiology of cognitive decline. ATP = Adenosine monophosphate, GTP = Adenosine guanosine monophosphate, AC = Adenylyl cyclase, GC = Guanylyl, PDE1 = Phosphodiesterase-1, 3ʹ,5ʹ-cAMP = 3ʹ,5ʹ-cyclic adenosine monophosphate, 5ʹ-cAMP = 5ʹ-cyclic adenosine monophosphate. ↑, increased sign; ↓, decreased sign.

Changes in neurotransmitter systems influence cognitive performance (Li et al., 2016). The omnipresent cyclic nucleotides cAMP and cGMP are upregulated during neuronal activation by ATP and GTP, respectively, and are inhibited by G inhibitory or stimulated by G stimulatory present on GTP binding protein (G-protein) coupled receptors by playing opposite key role (Heckman et al., 2017). Physiologically, when G alpha subunit (Gαs)-containing complexes stimulate AC, cAMP is produced. NO synthases synthesize NO. Consequently, NO provokes cytoplasmic soluble GC (sGC), and sGC synthesizes cGMP in the cytoplasm (Heckman et al., 2017; Sanders and Rajagopal, 2020). PDE1 hydrolyzes these second messengers into their inactive products and therefore terminates the signaling pathway. cAMP and cGMP have been found to be highly linked to motor and cognitive function and to regulating signal transduction and the synaptic spreading of different neurotransmitters in the brain (Shekarian et al., 2020a). The positive effect of cAMP/cGMP on CREB signaling decreases during AD pathogenesis. Evidence from neuropathological and preclinical studies shows that cAMP/PKA/CREB/BDNF and cGMP/PKG/CREB/BDNF pathways may decline pathologically in individuals with AD. In addition to PDE1, other mediators affect cAMP/cGMP signaling pathways. Human tau upregulation inactivates PKA, dephosphorylates CREB, and decreases the expression of BDNF mRNAs (Sanders and Rajagopal, 2020). Basal Gαs and forskolin reduce AC activity in the hippocampus in patients with AD. In cultured hippocampal neurons, Aβ expression inhibits PKA activity and glutamate-induced CREB phosphorylation (Sanders and Rajagopal, 2020). To date, reductions in synapses and neuronal injury are presumed to account for the decrease in neuroplasticity and increase in cognitive decline (Figure 4) (Heckman et al., 2014).

## 6 Vinpocetine as a selective PDE1 inhibitor

**FIGURE 5:**
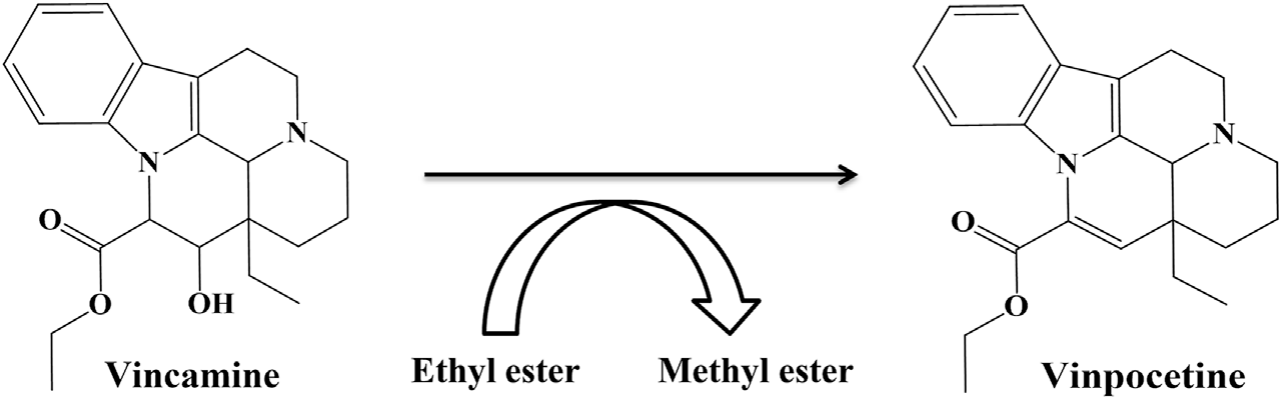
Structures of vincamine and vinpocetine.

Vinpocetine, a classical selective inhibitor of PDE1 (Heckman et al., 2017), is a dehydrated and semisynthetic derivative of the alkaloid vincamine derived from the family Apocynaceae, in which methyl ester is replaced with an ethyl ester (Al-Kuraishy et al., 2020; Karaer et al., 2022). Vinpocetine has been found to be more active than vincamine (Figure 5). Vinpocetine crosses the blood–brain–barrier (BBB) and enters the brain after oral or intravenous administration (Karaer et al., 2022). Vinpocetine is less soluble in water and more soluble in nonpolar vehicles (Ahad et al., 2022). Various clinical studies have proven the neuroprotective effects of vinpocetine (Karaer et al., 2022). Vinpocetine was developed and launched for the first time in Hungary in 1978 (Karaer et al., 2022) and marketed under the brand name Cavinton (Dubey et al., 2020). It has been extensively prescribed since. Vinpocetine has also been approved by the European and British pharmacopeias as an agent for the therapy of cognitive disorders (Dubey et al., 2020).

Neuroplasticity is the ability of a neuron to restore its structure, function, and connections after injury (Jellinger and Attems, 2013). Vinpocetine has been tested as a neuroplasticity promotor and marketed as a memory booster (Dubey et al., 2020).

Its memory-boosting effect is due to PDE1 inhibition, which increases cAMP and cGMP levels (Dubey et al., 2020). For more than 20 years, vinpocetin has been shown to reduce cognitive dysfunction in rodents and facilitate long-term potentiation linked to memory dysfunction. Additionally, in an intracerebroventricular streptozocin-induced rat model with AD-related cognitive symptoms, vinpocetine was found to restore memory function in the Morris water maze and passive avoidance test (Heckman et al., 2017).

In 2019, the Food and Drug Authority (FDA) stated warning against vinpocetine, reporting that during pregnancy, it can be harmful to the fetus or lead to miscarriage. Vinpocetine induces serious agranulocytosis. Anecdotally, several people have observed that the continuous intake of vinpocetine disturbs immune function. Commission E reported that the decreased immune function during the long-term usage of vinpocetine may lead to apoptosis. The prolonged ingestion of vinpocetine slightly reduces systolic and diastolic blood pressure, as well as serum glucose levels to some degree. Other reported adverse effects included nausea, flushing, dry mouth, dizziness, heartburn, headaches, and transient hypertension and hypotension (Dubey et al., 2020).

## 7 PDE1 inhibitors and AD

Functional recovery is likely the most important therapeutic effect to achieve because it greatly improves the quality of life. Functional recovery in AD implicates cognition enhancement (Heckman et al., 2014). Rolipram and caffeine were the first compounds that effectively restored cognitive deficits in animal models of AD by inhibiting PDE (García-Osta et al., 2012; Prickaerts et al., 2017; Sanders and Rajagopal, 2020).

 Despite the fact that in AD, certain modifications lead to the extreme expression of PDE1 that causes memory dysfunction. In contrary to this, some scientific data have proven, PDE1 inhibitors are specific to PDE1 enzymes, targeting them improve cognition and cure AD (Shekarian et al., 2020b). For example, deprenyl/selegiline inhibited PDE1A2 and caused the short-term amelioration of AD (Sanders and Rajagopal, 2020). Nimodipine is clinically useful for patients with subarachnoid hemorrhage and reduces the severity of cognitive deficits by targeting PDE1 (Ansari et al., 2019). However, the randomized clinical trials on these compounds often had inadequate sample sizes, methodological flaws, or short follow-up durations. Given this situation, vinpocetine has not been accepted by clinicians for universal routine use for any neurological disorder. A systematic review found promising yet inconclusive evidence favoring its use in patients with dementia (Panda et al., 2022). The discovery of a novel, natural, and nontoxic treatment for AD remains a great need (Nabavi et al., 2019). In the last decade, food and plant natural products have attracted growing interest due to their medical applications (Aydoğan, 2020). In addition, PDE1 inhibitors have potential uses for AD treatment owing to their potential neuroprotective role. Vinpocetine has been reported as a selective PDE1 inhibitor that can prevent the formation of inactive products from cAMP and cGMP. Overall, the balance between cAMP and cGMP levels is considered to be essential for shaping neuronal circuits (Delhaye and Bardoni, 2021). In addition to activating signaling pathways, these second messengers trigger the phosphorylation of α-amino-3-hydroxy-5-methyl-4-isoxazolepropionic acid receptors, which can normalize synapses and clear the way for glutamatergic transmission (Shekarian et al., 2020a). Vinpocetine for the treatment of memory dysfunction has entered phase IV clinical trials (ClinicalTrials.gov Identifier: NCT0O719953). In this phase, vinpocetine was evaluated as a nutritional supplement for alleviating cognitive impairments in elderly individuals. Although it showed memory-enhancing effects and improved cognition in healthy female participants, it was found to be ineffective in improving memory in patients with AD (Heckman et al., 2017).

IBMX, the first PDE1 inhibitor, is not very specific for PDE1 given that it also inhibits PDE5. Currently, PDE1 inhibitors with increased selectiveness have been identified, with ITI-214 being the furthest in development (Prickaerts et al., 2017). ITI-214, a newly developed PDE1 inhibitor, has a submicromolar binding tendency (>1000-fold) for all PDE1 isoforms (Wu et al., 2018b). In animal memory models, ITI-214 crosses the BBB and reaches the brain, where it exerts procognitive effects (O’Brien et al., 2020). A thienotriazolopyrimidinone PDE1 inhibitor (DNS-0056) with good pharmacokinetic and brain penetrative properties was developed recently. In a rat model of recognition memory, it significantly increased long-term memory without altering exploratory behavior (Wu et al., 2018b). Moreover, the previously discovered compound 3 m with a hydrophobic pocket has affinity for PDE1. The introduction of a hydrophobic group (e.g., benzyl group) into its pocket can further promote its PDE1 inhibitory activity (Huang et al., 2022). PF-04822163, a brain-penetrating quinazoline-based PDE1 inhibitor, was discovered by researchers from Pfizer. Compound SCH-51866 exhibited a selectivity of more than 300-fold for PDE1 over PDE5 (Dyck et al., 2017). (S)PF-04677940 has also been identified as a PDE1 inhibitor; however, detailed research on this compound remains unavailable (Huang et al., 2022). Moreover, DSR-141562, a well-known inhibitor of PDE1B, has been identified (Enomoto et al., 2019). It inhibits locomotor.

**FIGURE 6:**
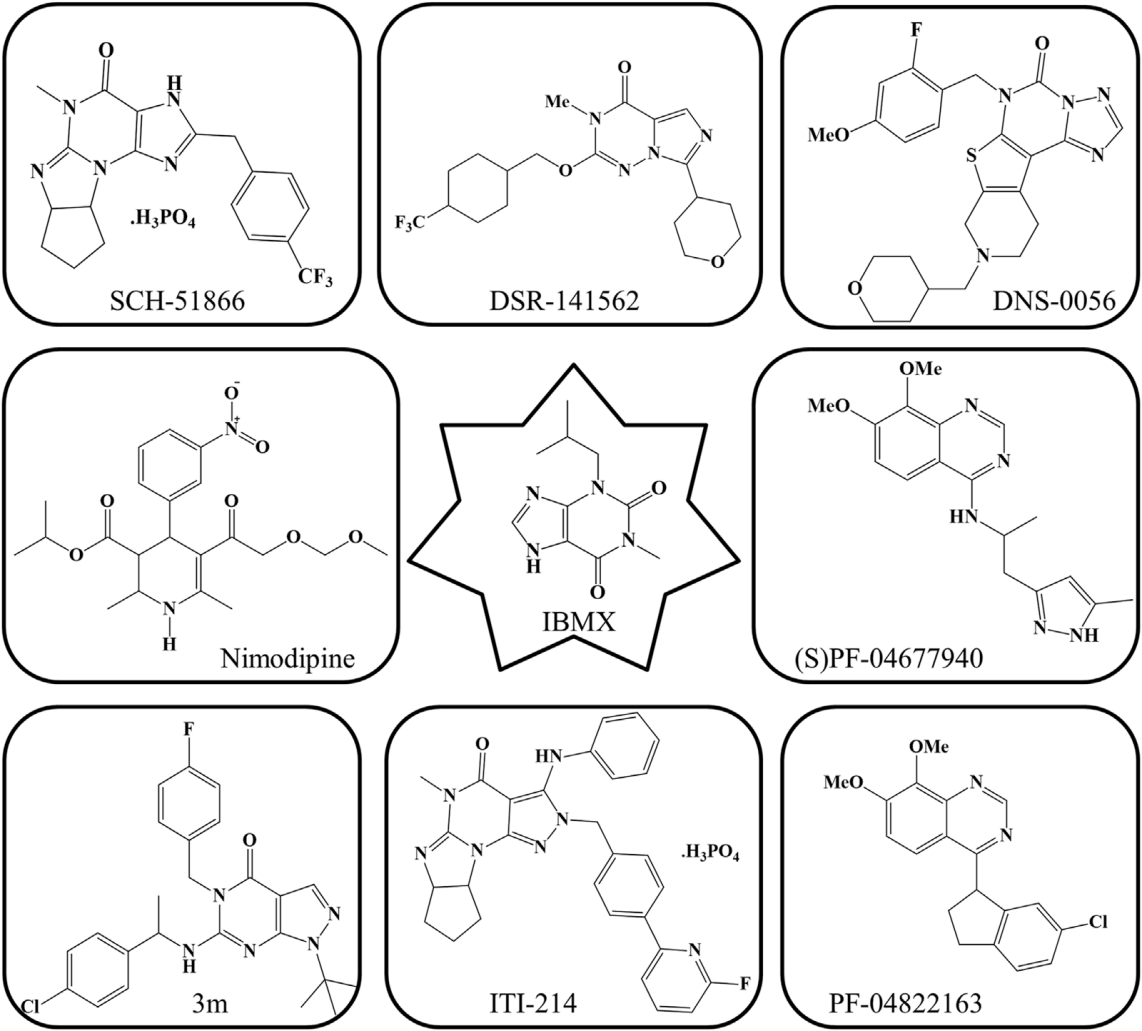
Current PDE1 inhibitors.

Hyperactivity and reverses dysfunctions in social interaction and novel object recognition in normal mice and rats (Delhaye and Bardoni, 2021). All PDE1 inhibitors, which have high affinity and penetrability, can have promising therapeutic effects on cognitive disorders and other conditions related to memory dysfunction (Dyck et al., 2017). The current PDE1 inhibitors, except for vinpocetine, which has been mentioned in Figure 5, are shown in Figure 6.

## 8 PDE1 inhibition and natural products

**FIGURE 7:**
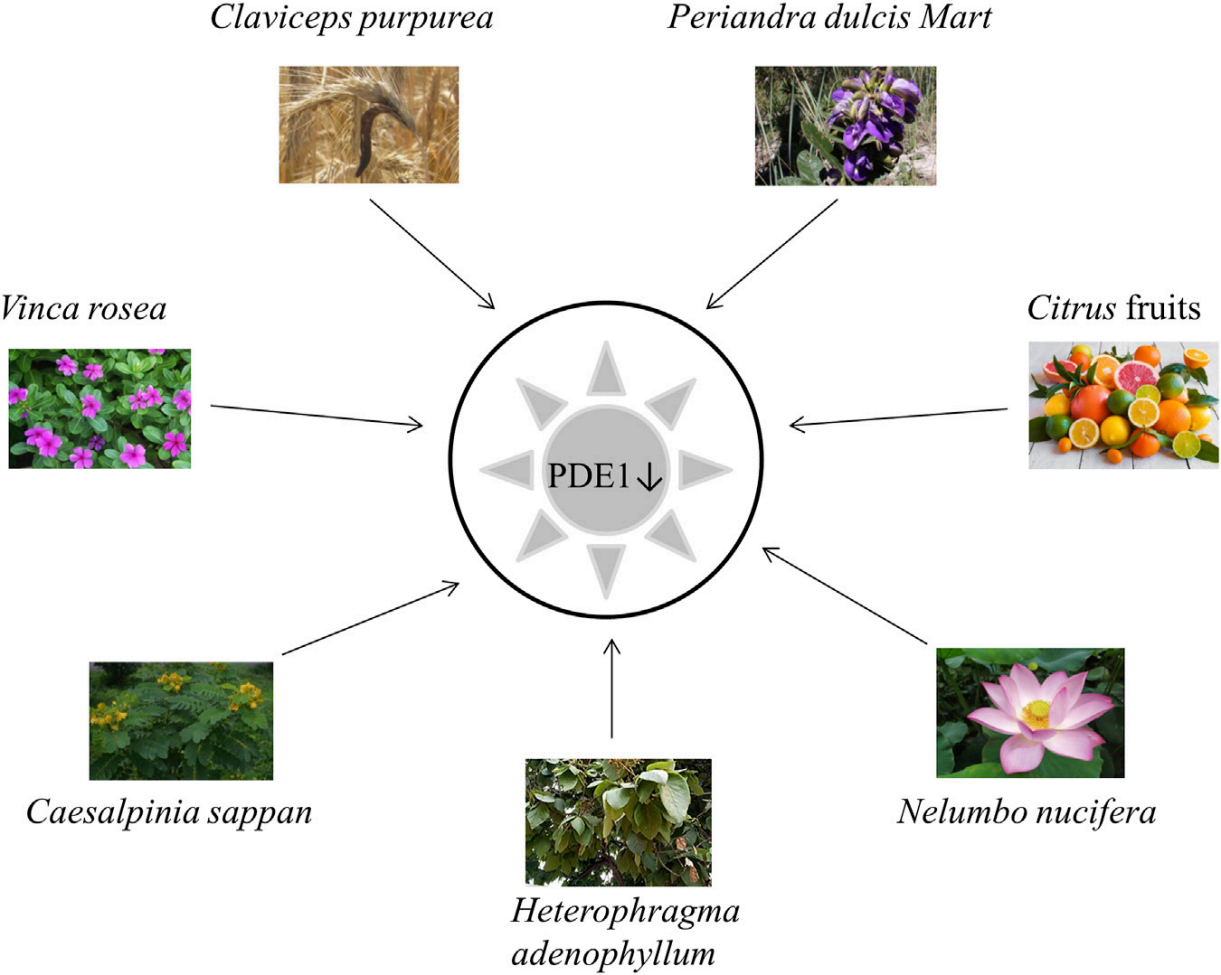
Plants inhibiting PDE1.

Herbal therapy is considered to be a very safe and approachable source of compounds for the treatment of NDDs (Dong et al., 2022). Numerous plants in the literature specifically target PDE1 in the brain (Helmi et al., 2020a). Certain plants have been found to be successful candidates for inhibiting the activity of PDE1 (Helmi et al., 2020b). Active compounds from plants that inhibit PDE1 are shown in Figure 7, and their details are given in Table 2.

**TABLE 2:** Detail of previously studied plant extracts as inhibitors of PDE1 and its isoforms.

Plant name	Herbal extract	Major compounds	Model	Target	Herbal extract dose	Durat-ion	*In vivo*/*In vitro*/In silico	Induced by	Route of admini-stration	Assessment	Action	References
*V. rosea*	Crude extract	Vinpocetine	Male adult Wistar rats	PDE1	4 mg/kg	30 days	*In vivo*	Amyloid-β peptide	Oral gavage	PAL, MWM,NOR	Antioxidative, learning and memory-enhancing effect	Shekarian et al. (2020a)
*C. sappan*	Ethanol extract	Brazilin	Mice	PDE1	250, 500 mg/kg	14 days	*In vivo*	Scopolamine	p.o	MWM	Memory-enhancing effect	Helmi et al. (2021)
	Ethanol extract	Brazilin	—	PDE1	10 μl of 100 μg/ml	—	*In vitro*	—	—	Assay	PDE1 inhibition	Helmi et al. (2020b)
	96% *n*-hexane, chloroform, and ethanol extracts	Brazilin	—	PDE1, PDE1B	100 μg/ml, 11.45 μg/ml, 14.02 μg/ml	—	*In vitro* and In silico	—	—	Assay	PDE1 inhibition	Helmi et al. (2020a)
*C. purpurea*	—	Nicergoline	—	PDE1	100 mcgM	—	*In vitro*	—	—	Assay	PDE1 inhibition	Sanders and Rajagopal, (2020)
*H. Adenophyllum* seem	Methanol extract	Compound-3	—	PDE1	8 mcg/L	—	*In vitro*	—	—	Assay	PDE1 inhibition	Shah et al. (2020)
	Methanol extract	Compound-3	—	PDE1B	—	—	In silico	—	—	Assay	PDE1 inhibition	Shah et al. (2020)
*N. nucifera*	—	Neferin	—	PDE1B	—	—	*In vitro*	—	—	Assay	PDE1 inhibition	Rahimi et al. (2010)
*Citrus* fruits	Purchased naringenin	(±)-Naringenin (flavanone)	—	PDE1	—	—	*In vitro*	—	—	Assay	PDE1 inhibition	Rahimi et al. (2010)
*P. dulcis* Mart	Methanol extract	Saponins (periandradulcins A,B,C)	—	PDE1	—	—	*In vitro*	—	—	Assay	PDE1 inhibition	Rahimi et al. (2010)

mg, milligram; µg/mcg, microgram; g, gram; ml, milligram; M, molar; µL, microlitre; L, litre, p.o, per oral; PDE1, Phosphodiesterase-1.

Only a few PDE1 inhibitors are available on the market. Vinpocetine, a PDE1 inhibitor, remains widely used for the treatment of dementia in AD. However, it has some undesirable side effects as already mentioned in the section “Vinpocetine as a selective PDE1 inhibitor.” Therefore, finding alternative PDE1 inhibitors, especially those from natural plant sources, is still needed (Helmi et al., 2020a). Natural extracts from plants and their bioactive compounds are considered essential due to their safe and promising clinical results. Interestingly, during the last decade, several extracts from plants have been shown to exhibit significant PDE inhibitory potential. Several natural compounds from plants have been demonstrated to have cAMP or cGMP-specific or dual specificity. In traditional practices, various plants, such as *V. rosea*, *C. sappan L.*, *C. purpurea*, *P. dulcare* Mart, *H. adenophyllum* Seem, *N. nucifera* and citrus fruits, have been applied to treat cognitive disorders, such as AD (Kumar et al., 2015).

*Catharanthus roseus* L. (also known as *V. rosea*) is a perennial plant that is mostly found in Southern Asia and tropical countries. It is also native to Madagascar. It is locally known in Malaysia as *kemunting cina*. This plant has many common names, such as bright eyes, Madagascar periwinkle, graveyard plant, Cape periwinkle, old maid, rose periwinkle myrtle, and pink periwinkle. It is used for ornamental purposes because of its various colors, such as pink, purple, and white. It is highly popular due to its medicinal applications. This plant is used in chemotherapeutic regimes to treat cancer and childhood leukemia, hypertension, diabetes, malaria, nonsmall lung cancer, and Hodgkin’s lymphoma and to improve memory. It has antioxidant, antimicrobial, hypolipidemic, antidiarrheal, and wound-healing activities (Allamsetty et al., 2020). A recent study mentioned that *V. rosea* also has PDE1 inhibitory activity (Table 2) (Shekarian et al., 2020a).

*C. sappan* (local name *secang*), is a medicinal plant that is widely used as a natural red dye, herbal drink, and traditional herbal medicinal preparation in Yogyakarta, Indonesia. Numerous reports have shown that *C. sappan* has antioxidant, antibacterial, antidiarrheal, antiinflammatory, anticancer, hepatoprotector, and antidiabetic activities. *C. sappan* has been scientifically documented to enhance cognition *via* PDE1 inhibition (Table 2) (Helmi et al., 2020a; Helmi et al., 2020b).

*C. purpurea*, commonly known as ergot fungi, occurs predominantly in the northern temperate zone (Pažoutová et al., 2015). It is probably the most widely cultivated fungus and has become an important field crop because it contains ergot alkaloids that are extensively used as medicine. Ergot alkaloids have a wide range of therapeutic uses as a highly potent drug for the treatment of *postpartum* bleeding, uterine atonia, migraine, senile cerebral insufficiency, orthostatic circulatory disturbances, hypertension, hyperprolactinemia, acromegaly, and Parkinson’s disease; they also have immunomodulatory and hypolipemic activities (Křen et al., 1994). The ability of this plant to inhibit PDE1 has been scientifically recorded by recent studies (Table 2) (Sanders and Rajagopal, 2020).

*H. adenophyllum* Seem, commonly known as *zihhaw* or *marodphali katsagon*, is found in Delhi and distributed in Africa and Southeast Asia. It has multiple medicinal applications in traditional Thai medicine systems and various biological activities, including antimicrobial, antidiabetic, antiseptic, antiendemic, antiabscess, antiulcer, antiinflammatory, antimalarial, anticarcinomic, viricidal, and termiticidal activities. It is also used to treat amenorrhea and constipation. The chemical compounds from *H. adenophyllum* demonstrated significant PDE1 inhibitory activity that reflected the role of the secondary metabolites in the inhibition of vasoconstriction and inflammation (Table 2) (Shah et al., 2020).

*N. nucifera* (common name: lotus) is widely cultivated in North Australia, Asia, Egypt, and the Caspian Sea (Ahn et al., 2014). This plant has a long history of usage as a folk herbal medicine in China (Wu et al., 2020). Its rhizome has long been used as a food source and treatment for diarrhea, hemorrhage, constipation, and AD and to improve learning and memory behavior (Ahn et al., 2014). In recent decades, lotus has attracted growing attention from the scientific community. An increasing number of research papers focusing on *N. nucifera* have been published and shed light on the mysteries of this species. Lotus is an important horticultural plant that is also commonly used for ornamental, nutritional, and medicinal purposes (Lin et al., 2019). Numerous studies have demonstrated that *N. nucifera* confers various kinds of health benefits, including antithrombotic, antitumor, antioxidative, antidiabetic, antiarrhythmic, inflammatory, and neuroprotective effects and prevents cellular death and damage in hyperglycemia-induced endothelial cells. Moreover, accumulating evidence indicates that this plant imparts neuroprotective effects to the CNS by inhibiting oxidative stress, apoptosis, and neuroinflammatory processes (Table 2) (Wu et al., 2020).

In addition, essential micronutrients, e.g., vitamin C (from citrus fruits), represents a rich source of nonessential bioactive compounds, particularly flavanones. Numerous preclinical studies have demonstrated that citrus flavonoids have neuroprotective potential, antioxidative and antiinflammatory activities, and mechanistic actions on BBB function or integrity. Therefore, scientists recommend encouraging the consumption of citrus fruits in the form of whole fruit and 100% juices for their potential neurological benefits (Braidy et al., 2017; Pontifex et al., 2021). Current pilot clinical research has indicated that treatment with nobiletin-rich *Camellia reticulate* peel extracts decelerate decline in patients with AD treated with donepezil without adverse effects (Braidy et al., 2017). Rahimi et al. reported that citrus fruits contain bioactive compounds potent inhibitors of PDE, especially PDE1 (Table 2) (Rahimi et al., 2010).

*P. dulcis* Mart*.* (accepted name: *Periandra mediterranea*) is native to the northern and middle parts of Brazil and is used in Brazilian ethnomedicine. It performs numerous biological activities, such as expectorant, diuretic, antiinflammatory, laxative, antifungal, antiplatelet aggregation, cytotoxic, and hemolytic activities; inhibits tumor cell proliferation; and lowers blood cholesterol and triacylglycerol levels (Negri and Tabach, 2013). The literature showed that *P. dulcis* Mart. can inhibit PDE1 (46). Yoshitaka Ikeda et al. (1991) reported that compound 1 from this plant is the most potent known PDE inhibitor and inhibited PDE1 20–40 times more effectively than PDE2 and PDE3 (Table 2) (R. Iwahori, 1970).

## 9 Ongoing updates on PDE1 inhibition in AD

Only a few ongoing investigations on PDE1 inhibitors were found (Perneczky, 2019). The progress in the development of novel 1,2,3-triazole and 1,2,4-triazole planned molecules as drugs with various molecular targets in AD, such as PDE1 inhibitors, was reported by Prasanna and Sharma (Prasanna and Sharma, 2022). ITI-214 is being considered for clinical development (Prickaerts et al., 2017). IBMX (Prickaerts et al., 2017), DNS-0056 (Wu et al., 2018b), PF-04822163 (Dyck et al., 2017), 3 m (Huang et al., 2022), SCH-51866 (Dyck et al., 2017) and (S)PF-04677940 are compounds with PDE1 inhibitory activity (Huang et al., 2022). ITI-214 and SCH-51866 have shown activity in the brain, whereas other compounds inhibit PDE1 only in the lung and heart (Dyck et al., 2017; Prickaerts et al., 2017). In a cross-sectional multicenter study on 187 patients with major depressive disorder, quetiapine was prescribed to augment first-line AD psychopharmacotherapy and revealed large-scale beneficial effects (Bartova et al., 2022). In addition to PDE1 inhibitors, aducanumab, a monoclonal antibody, was approved by the United States in June 2021 as a first novel putative disease-modifying therapy against Aβ in the brain. However, only four anti-AD drugs are currently used to mask dementia symptoms, among which three are cholinesterase inhibitors and one is memantine (Nagata et al., 2022). These compounds and drugs have been considered as suitable targeting species for PDE1.

## 10 Limitations

Recent studies on PDE1 clearly show some limitations that affect AD therapy. The major issue is the nonspecificity of vinpocetine for other receptors, showing low target protein selectivity (Roks, 2022). Few persistent clinical studies have been carried out on vinpocetine (Heckman et al., 2017). Until now, the FDA has not approved vinpocetine for the treatment of cognitive dysfunction although it is currently used as a cognitive enhancer to treat memory impairments. However, the possible beneficial effect of vinpocetine on cognition remains questionable given that vinpocetine did not exhibit positive effects on memory function in healthcare centers (Heckman et al., 2017). Give that PDE1 is not only present in the brain but is also present in other organs, such as the heart and lungs, hence, finding the most selective PDE1 inhibitor remains necessary (Heckman et al., 2017). No authentic laboratory rodent AD model has been established. In addition, AD has been found to be linked to changes and genetic diseases. However, genetic variations in AD are still ignored. This situation is a remarkable limitation that must be evaluated in future investigations (Shekarian et al., 2020a).

## 11 Conclusion and later prospects

The exploration of PDE1 inhibitors remains crucial for the development of novel substitutes for anti-AD drugs (Harfouche et al., 2022). This review focuses on developed PDE1 inhibitors (Sadiqa et al., 2021). To the best of our knowledge, only a few PDE1 inhibitors have been evaluated in preclinical AD animal models as mentioned in this review (Heckman et al., 2017). Among these inhibitors, vinpocetin is in phase IV (Heckman et al., 2017) and ITI-214 is in phase II (Wu et al., 2018b) trials. We found some study gaps. For example, the synergy between *Cesalpinea shuppan* and PDE1 inhibition requires further studies (Helmi et al., 2020b). Nimodipine is inadequate for use as a long-term memory booster despite its short-term advantages in vascular dementia. These effects were found in geriatrics who were affected by vascular dementia in the subcortical region and need to be elucidated in large-scale trials with other groups (Tomassoni et al., 2008). A double-blinded study recommended the routine prescription of vinpocetine; however, vinpocetine has not progressed to placebo-controlled randomized clinical trials with a particular sample size (Panda et al., 2022). Vinpocetine remains extensively used as a nootropic owing to its memory-boosting effect, which may be correlated with its vasodilating effect. However, this therapeutic property of vinpocetine remains disputed (Prickaerts et al., 2017). The employment of natural compounds or their derivatives that target PDE1 specifically as possible AD treatments can support and promote traditional therapy and open new paths for the development of natural plant products with increased efficiency (Dubey et al., 2020; Sadiqa et al., 2021). Certain therapeutic components from plants, including melatonin, resveratrol, and curcumin, have been identified as natural AD treatments. However, these bioactive compounds exhibit unsatisfactory bioavailability because of their aqueous solubility, metabolic issues, and permeability. Hence, additional investigations are required to accept these compounds as therapeutic agents for AD treatment (Sadiqa et al., 2021). Plants have also been found to have PDE inhibitory activity, but which product shows the best PDE1 inhibitory effect on different neuronal injuries still needs to be verified. Only few development strategies for AD treatment exist (Sadiqa et al., 2021). Nutraceuticals have been advertised in the medical field. The ingestion of nutraceuticals with meals is encouraged to reduce AD risk. However, nutraceuticals are very far from attracting as much attention as allopathic medicine due to some restrictions, such as the lack of clinical data. Nevertheless, their neuroprotective potential in cognitive dysfunction must be explored deeply (Sadiqa et al., 2021). Undoubtedly, clinical trials on herbal preparations for the treatment of patients with AD are in progress and may improve the future of memory impairment in AD (Delhaye and Bardoni, 2021). In the near future, we may see many compounds from natural sources undergo clinical trials and emerge as novel beneficial agents against AD, ultimately improving cognitive dysfunction.
